# Local Anesthesia for Complex F/BEVAR in a High-Risk Cohort: A Single-Center Feasibility Study

**DOI:** 10.3390/jcm15093257

**Published:** 2026-04-24

**Authors:** Natasha Hasemaki, Ihza Fachriza, Jan Stana, Alexia-Vasiliki Amvrazi, David Khangholi, Tugce Öz, Nikolaos Konstantinou, Nikolaos Tsilimparis

**Affiliations:** 1 Department of Vascular Surgery, University Hospital LMU Munich, Marchioninistr. 15, 81377 Munich, Germany; natasha.hasemaki@med.uni-muenchen.de (N.H.); ihzafachriza@gmail.com (I.F.); jan.stana@med.uni-muenchen.de (J.S.); alexia.amvrazi@med.uni-muenchen.de (A.-V.A.); david.khangholi@med.uni-muenchen.de (D.K.); tugce.oz@med.uni-muenchen.de (T.Ö.); nikolaos.konstantinou@med.uni-muenchen.de (N.K.); 2 Division of Vascular and Endovascular Surgery, Department of Surgery, Faculty of Medicine, Universitas Indonesia, Jakarta 10430, Indonesia; 3 Division of Vascular and Endovascular Surgery, Clinical Department of Cardiovascular, Cipto Mangunkusumo National Hospital, Jakarta 10430, Indonesia

**Keywords:** complex aortic, fenestrated EVAR, branched EVAR, local anesthesia

## Abstract

**Background/Objectives**: Fenestrated and branched endovascular aortic repair (F/BEVAR) is increasingly used for the treatment of complex aortic aneurysms, and is traditionally performed under general anesthesia (GA). Data on the use of local anesthesia (LA) for F/BEVAR remain limited. This study aimed to report early outcomes of F/BEVAR performed under LA versus GA, with a focus on feasibility and perioperative complications in a high-risk patient population. **Methods**: This single-center retrospective analysis included patients undergoing F/BEVAR under LA or GA. Primary outcomes were in-hospital mortality and in-hospital complications. Secondary outcomes included early reintervention, intensive care unit and hospital length of stay, blood transfusion requirements, and technical success. **Results**: A total of 359 patients were included, of whom 25 (7.0%) were treated under LA and 334 (93.0%) under GA. Conversion from LA to GA occurred in 6 patients (24%). Patients in the LA group represented a higher-risk cohort, with advanced age, higher ASA class, larger aneurysm diameters, and a greater proportion of emergency and ruptured repairs. Technical success was high, and procedural metrics were within expected ranges. In-hospital mortality was numerically higher in the LA group (12.0% vs. 2.9%, *p* = 0.05). Overall, in-hospital complications were more frequent in the LA group (68.0% vs. 41.3%, *p* = 0.009), including a higher rate of spinal cord ischemia (24.0% vs. 8.5%, *p* = 0.02). Blood transfusion requirements were also greater in patients treated under LA (*p* = 0.004), while blood loss, ICU stay, and hospital length of stay were comparable. Early reintervention occurred more frequently in the LA group (31.8% vs. 10.4%, *p* = 0.009). **Conclusions**: LA appears feasible in selected high-risk patients undergoing complex F/BEVAR. However, given substantial baseline differences between groups, no conclusions can be drawn regarding comparative safety or efficacy relative to GA. These findings should be considered preliminary.

## 1. Introduction

The treatment paradigm for complex abdominal aortic aneurysms (cAAAs) and thoracoabdominal aortic aneurysms (TAAAs) has increasingly shifted toward endovascular repair, which has become the predominant treatment approach in most tertiary referral centers [1,2,3,4]. Despite being less invasive than open surgery, fenestrated and/or branched endovascular aortic repair (F/BEVAR) remains technically demanding, often requiring prolonged operative times and strict patient immobility.

Consequently, general anesthesia (GA) has traditionally been the anesthetic technique of choice for F/BEVAR procedures. However, GA is associated with recognized risks, particularly in elderly patients and those with multiple comorbidities, including respiratory complications, increased cardiopulmonary stress and prolonged postoperative recovery [5,6,7]. Beyond these perioperative complications, induction of GA in urgent settings may precipitate acute hemodynamic deterioration, with the potential conversion of a contained into a free rupture [8]. In contrast, local anesthesia (LA) is well established for standard endovascular aortic repair (EVAR) and has been associated with reduced cardiopulmonary morbidity and shorter length of hospital stay. Accordingly, current European Society for Vascular Surgery (ESVS) guidelines recommend LA for EVAR in ruptured abdominal aortic aneurysms (AAAs) when feasible, in order to avoid the hemodynamic consequences of GA induction [9].

The role of LA in F/BEVAR, however, remains insufficiently investigated. Although limited evidence suggests that LA may be feasible in carefully selected patients, its potential advantages may be particularly relevant in this setting. These include attenuation of physiological stress and the ability to maintain patient interaction during complex endovascular procedures, with the possibility of real-time neurological assessment during extensive aortic coverage, which may facilitate early detection of spinal cord ischemia, one of the most serious complications of complex aortic repair. Nevertheless, concerns persist regarding patient tolerance during prolonged procedures and the overall safety of LA in complex or urgent F/BEVAR cases. While GA remains the standard approach, current practice varies in selected high-risk patients, largely reflecting institutional experience and individualized clinical assessment in the absence of robust data to guide anesthetic selection. This study aimed to evaluate the feasibility of LA in selected high-risk patients undergoing F/BEVAR and to describe associated clinical outcomes, rather than to directly compare anesthesia strategies.

## 2. Materials and Methods

### 2.1. Study Design and Population

This study was a single-center retrospective cohort analysis of prospectively collected data from consecutive patients undergoing F/BEVAR procedures for TAAAs or CAAAs. All patients were treated at University Hospital LMU Munich between 1 September 2018 and 31 December 2025.

Patients were stratified according to anesthetic management into LA or GA groups. The choice between local and general anesthesia was made on a case-by-case basis by the treating vascular surgeon and anesthesiologist. GA represents the standard approach for F/BEVAR at our institution. LA was selectively considered in patients at high risk for GA, particularly in the setting of advanced age, significant cardiopulmonary comorbidity, or urgent or ruptured presentations. Contraindications to LA included inability to cooperate, severe anxiety, or anticipated procedural complexity requiring GA. Pre-, intra-, and postoperative data, after pseudonymization, were collected in a local database. The study adhered to the STROBE (STrengthening the Reporting of OBservational Studies in Epidemiology) guidelines [10]. The study was conducted in accordance with the Declaration of Helsinki and deemed exempt from ethical approval due to its retrospective design and use of anonymized data.

### 2.2. Data Collection

Baseline demographic and clinical variables included age, sex, body mass index (BMI), American Society of Anesthesiologists (ASA) physical status classification, and major cardiovascular, pulmonary, renal and metabolic comorbidities. Previous aortic surgical history was documented, including prior open AAA repair, EVAR, thoracic endovascular aortic repair (TEVAR), and frozen elephant trunk (FET) procedures. Procedural characteristics included urgency of repair (elective vs. emergency), rupture status, maximum aneurysm diameter, access strategy (percutaneous vs. surgical cutdown), upper extremity access, number of target vessels (TVs), operative time, and technical success.

### 2.3. Anesthetic Strategy

In the LA group, procedures were performed using local anesthetic infiltration at vascular access sites, combined with conscious sedation as required. Sedation was administered and titrated by the attending anesthesiologist using short-acting agents to maintain patient comfort while preserving responsiveness to verbal stimuli. Standard intraoperative monitoring was applied in all patients, including continuous hemodynamic and respiratory monitoring. Airway support equipment and personnel were immediately available, and conversion to GA was performed at the discretion of the anesthesiologist and surgical team in cases of patient intolerance, hemodynamic instability, or procedural complexity.

In the GA group, procedures were performed under GA with endotracheal intubation and standard invasive monitoring according to institutional protocols.

### 2.4. Definitions and Outcomes

The primary outcomes were in-hospital mortality and in-hospital complications. In-hospital mortality was defined as death from any cause occurring during the index hospitalization. In-hospital complications comprised any major postoperative complication occurring during hospitalization, including cardiopulmonary, neurological, renal, infectious, gastrointestinal, bleeding-related, or access-related events. Spinal cord ischemia (SCI) was defined as any new postoperative motor or sensory deficit consistent with spinal cord injury. Renal failure was defined as new-onset renal dysfunction requiring dialysis or a significant increase in serum creatinine. Cardiopulmonary complications included events such as myocardial infarction, heart failure, arrhythmia, or pneumonia. Access-related complications included bleeding, vascular injury, or need for surgical revision at the access site. The composite endpoint of in-hospital complications included clinically relevant postoperative complications, as defined above.

Secondary outcomes included early reintervention, intensive care unit (ICU) length of stay, total hospital length of stay, estimated blood loss, blood transfusion requirement and technical success. Early reintervention was defined as any secondary endovascular or surgical procedure performed within 30 days postoperatively or during the index hospitalization and included procedures for endoleak management, target vessel-related issues, access complications, or treatment of postoperative complications. Technical success was defined as successful deployment of the endograft with catheterization of all intended target vessels and absence of major intraoperative complications, in line with standard reporting practices. Immediate intervention was recorded as well and was defined as any unplanned surgical or endovascular procedure performed intraoperatively or within 24 h of the index procedure.

### 2.5. Statistical Analysis

Continuous variables were assessed for normality using the Shapiro–Wilk test. Normally distributed data are presented as mean ± standard deviation (SD) and were compared using the independent samples Student’s *t*-test. Non-normally distributed variables are presented as median with interquartile range (IQR) and were compared using the Mann–Whitney U test. Categorical variables are presented as frequencies and percentages and were compared using the chi-square test or Fisher’s exact test, as appropriate. For variables with missing data, percentages were calculated using available-case denominators for each variable. All tests were two-sided, and a *p*-value < 0.05 was considered statistically significant. Standardized mean differences were calculated for baseline variables to quantify imbalance between groups. For key binary outcomes, odds ratios with 95% confidence intervals were calculated descriptively using available-case data. Given the observational design and baseline differences between groups, analyses were considered exploratory and intended to describe associations rather than establish causality. All statistical analyses were performed using IBM SPSS Statistics (version 31.0; IBM Corp., Armonk, NY, USA).

## 3. Results

During the study period, a total of 359 patients underwent F/BEVAR for TAAA or CAAA at our institution. Of these, 25 patients (7.0%) were treated under LA and 334 patients (93.0%) under GA. Six patients (24%) required intraoperative conversion to general anesthesia, including one due to patient intolerance, three due to hemodynamic instability requiring anesthetic control, and two due to increasing procedural complexity. Conversion most commonly occurred during the main endovascular phase of the procedure. Patients treated under LA represented a higher-risk cohort compared with those treated under GA (Table 1). The LA group was significantly older (78.2 ± 9.6 vs. 72.1 ± 9.5 years, *p* = 0.002), with a higher proportion of patients aged >75 years (72.0% vs. 42.5%, *p* = 0.004), and a greater prevalence of ASA class IV–V (56.0% vs. 20.7%, *p* < 0.001). Standardized mean differences demonstrated substantial baseline imbalance between groups, particularly for age, ASA class, and prior aortic interventions.

Procedural characteristics differed between groups, reflecting greater complexity in patients treated under local anesthesia (Table 2). The distribution of aneurysm type did not differ significantly between groups (*p* = 0.14). However, emergency procedures (64.0% vs. 27.7%, *p* < 0.001) and repairs for ruptured aneurysms (52.0% vs. 10.5%, *p* < 0.001) were significantly more frequent in the LA group. Aneurysm diameter was larger in the LA group (64.5 [59.0–88.5] mm vs. 60.0 [54.0–70.0] mm, *p* < 0.001). With regard to procedural strategy, the type of endovascular repair differed significantly between groups (*p* < 0.001). Within the LA group, the majority of procedures were BEVAR (72.0%), whereas FEVAR was more commonly performed in the GA group (55.7%). Procedural complexity was further reflected by a higher number of target vessels in the LA group (4.2 ± 0.8 vs. 3.9 ± 0.5, *p* = 0.018), as well as a greater proportion of procedures involving more than four target vessels (16.0% vs. 4.5%, *p* = 0.035). Upper extremity access was also more frequently required in the LA group (66.7% vs. 35.6%, *p* = 0.008). Additional procedures were significantly more common in the LA group (78.9% vs. 37.0%, *p* < 0.001), whereas staged repair (0.0% vs. 40.2%, *p* < 0.001) and MISACE (0.0% vs. 23.1%, *p* = 0.008) were performed more frequently in the GA group. The use of cerebrospinal fluid drainage was also higher in the GA group (28.0% vs. 4.2%, *p* = 0.01). Percutaneous lower extremity access was similarly high in both groups (95.5% vs. 95.7%, *p* = 1.00), and no differences were observed in the use of custom-made devices (*p* = 0.48). Standardized mean differences demonstrated substantial imbalance in procedural characteristics, particularly with respect to rupture status, emergency presentation, type of endovascular repair, use of staged repair, and neuroprotective strategies.

Technical success was high in both groups (96.0% vs. 94.3%, *p* = 1.00). Operative time, fluoroscopy time, and contrast dose were also similar between groups. In-hospital mortality was numerically higher in the LA group (12.0% vs. 2.9%; OR 4.92, 95% CI 1.24–19.47), although this finding should be interpreted with caution given the small sample size, wide confidence intervals, and substantial baseline imbalance (*p* = 0.05). Detailed causes of death were not consistently available. However, overall in-hospital complications were significantly more frequent in the LA group (68.0% vs. 41.3%, OR 3.33, 95% CI 1.35–8.19, *p* = 0.009). Spinal cord ischemia occurred more frequently in the LA group (24.0% vs. 8.5%, OR 3.74, 95% CI 1.37–10.19, *p* = 0.02), while no significant differences were observed in other individual complications, including cardiopulmonary, renal, or access-related events. Patients treated under LA required significantly greater blood transfusion volumes (316.0 [0.0–723.0] vs. 0.0 [0.0–500.0], *p* = 0.004), whereas blood loss was comparable between groups (*p* = 0.55). ICU stay and total hospital length of stay did not differ significantly between groups (*p* = 0.42 and *p* = 0.08, respectively). Early reintervention within 30 days occurred more frequently in the LA group (31.8% vs. 10.4%, OR 4.02, 95% CI 1.57–10.30, *p* = 0.009). Reinterventions were heterogeneous and included procedures for endoleak management, target vessel-related issues, access-related complications, and treatment of postoperative complications. Outcomes are summarized in Table 3.

## 4. Discussion

In this single-center observational study, we evaluated the feasibility and early outcomes of F/BEVAR performed under LA in a selected high-risk cohort, in the context of a predominantly GA-treated population. Patients treated under LA represented a markedly higher-risk cohort, characterized by advanced age, higher ASA classification, larger aneurysm diameters, and a substantially greater proportion of emergency and ruptured repairs. In addition, procedural characteristics suggested increased complexity in the LA group, including a higher number of target vessels, more frequent upper extremity access, and a greater need for adjunctive procedures. In this context, technical success was high and procedural metrics, including operative time, fluoroscopy time, and contrast usage, were within expected ranges. Conversion from LA to GA occurred in six cases, underscoring that LA is not universally feasible. Common reasons for conversion include patient intolerance of prolonged immobility, hemodynamic instability requiring advanced support, and unexpected procedural complexity requiring extended operative time. This finding highlights the importance of careful patient selection, anticipation of procedural complexity, and the need for immediate availability of anesthetic support to allow safe and timely conversion when required.

In-hospital mortality was numerically higher in the LA cohort, although this difference did not reach statistical significance. The observed difference in mortality should be interpreted with caution given the small size and wide confidence intervals. Overall in-hospital complications were more frequent in patients treated under LA. This finding must be interpreted in the context of substantial baseline differences between groups. The LA group included a greater proportion of urgent and ruptured repairs, which is also reflected in larger aneurysm diameter and higher blood transfusion requirements. Importantly, no significant differences were identified in rates of individual cardiopulmonary, renal, or access-related complications. These findings are likely influenced by differences in baseline risk profile and procedural characteristics rather than anesthetic strategy.

A notable finding of this study was a difference in the incidence of SCI between groups. This finding is likely multifactorial and should be interpreted in the context of substantial differences between groups. Patients treated under LA more frequently underwent emergency and ruptured repairs and were less likely to receive adjunctive protective strategies, including staged repair and MISACE. In addition, cerebrospinal fluid drainage was used less frequently in the LA group. Together, these factors reflect differences in clinical presentation and procedural strategy and may contribute to the observed difference in SCI rates. These findings highlight the importance of neuroprotective strategies, including staged repair when feasible, in reducing the risk of SCI in complex aortic repairs.

Early reintervention rates were higher in the LA group. This finding may reflect the higher proportion of urgent and complex cases in this cohort. Reinterventions in this study were heterogeneous, including both access-related procedures, adjunctive endovascular corrections, and treatment of postoperative complications. As such, reintervention should not be interpreted solely as a marker of procedural failure, but rather as part of the overall management of a high-risk patient population.

The role of LA in urgent endovascular repair is well established in the context of EVAR [5,6,7,8,11,12]. Large registry analyses and observational studies have demonstrated favorable early outcomes for ruptured and symptomatic AAAs treated under LA, including comparable or lower short-term mortality and reduced cardiopulmonary complications compared with GA. On this basis, current ESVS guidelines recommend LA for endovascular repair of ruptured AAAs whenever feasible [9].

In contrast, evidence supporting the use of LA in complex endovascular repair involving F/BEVAR remains limited [13,14,15,16]. Most available data are restricted to small series and case reports, reflecting the technical complexity of these procedures and the traditional reliance on GA for optimal procedural control. The largest dedicated case series to date, reported by Abisi et al., included 44 consecutive patients undergoing F/BEVAR under LA, predominantly for TAAAs requiring extensive aortic coverage [13]. Despite the procedural complexity and prolonged operative times, 91% of cases were completed without conversion to GA, no patients developed SCI, and no prophylactic cerebrospinal fluid drainage was required. Thirty-day mortality was 0%, and early neurological outcomes were favorable. A key contribution of this series was the demonstration that continuous intraoperative neurological assessment is feasible during F/BEVAR and may represent an alternative neuroprotective strategy to routine spinal drainage.

In addition, isolated case reports have described adjunct techniques to facilitate F/BEVAR procedures under LA in patients with prohibitive anesthetic risk [14,15]. The use of hypnosis or immersive virtual reality has been reported as a non-pharmacological adjunct to improve patient tolerance during prolonged endovascular procedures. Although limited to single-patient experiences, these reports highlight adaptive strategies that may enhance procedural feasibility in highly selected patients [14,15].

Experience from TEVAR further supports the feasibility of non-general anesthetic strategies in complex aortic interventions [17,18]. TEVAR performed under LA has been shown to be feasible with acceptable early outcomes, particularly in urgent settings and in patients at high anesthetic risk. Although TEVAR differs anatomically and technically from F/BEVAR, these data provide supportive evidence that complex endovascular aortic procedures can be performed without general anesthesia in selected patients at experienced centers.

The ability to perform awake neurological monitoring during complex aortic repair represents a theoretical advantage of LA [13]. SCI remains a feared complication of extensive aortic coverage, particularly in TAAA repairs [19]. Awake patient interaction allows immediate recognition of neurological symptoms during the procedure, enabling prompt corrective measures such as blood pressure optimization, modification of procedural strategy, or staged intervention before irreversible injury occurs. While indirect neuromonitoring strategies are available under GA, awake neurological assessment provides a direct and immediately interpretable feedback that may be particularly valuable in high-risk patients [20].

Additional potential advantages of LA include avoidance of hemodynamic fluctuations associated with induction and emergence from general anesthesia, preservation of spontaneous ventilation with reduced effects on venous return and cardiac output, attenuation of systemic inflammatory response, immediate neurological assessment, and faster recovery and mobilization [21,22].

Despite these potential advantages, LA also presents specific challenges. The theoretical benefits of awake monitoring must be balanced against the difficulties of reliably assessing neurological function in sedated patients and the potential for patient anxiety if neurological deficits are detected intraoperatively. In addition, anxiety, pain, or discomfort may provoke sympathetic activation with hypertension and tachycardia, whereas vasovagal responses or sedation-related respiratory depression may result in hypotension and bradycardia. Successful use of LA therefore requires careful patient selection, meticulous titration of analgesia and sedation, continuous hemodynamic monitoring, and close collaboration between surgical and anesthesia teams. Patient psychological profile, pain tolerance, and ability to remain immobile for prolonged periods should be considered during preoperative planning. In the absence of robust comparative studies, firm conclusions regarding superiority or equivalence between anesthetic strategies in complex endovascular repair cannot be drawn.

Overall, these findings indicate that the use of LA in F/BEVAR is feasible in carefully selected patients. However, given the substantial baseline and procedural differences between groups, no conclusions can be drawn regarding its comparative safety or efficacy relative to GA.

## 5. Limitations

This study has several important limitations that should be considered when interpreting its findings. First, its retrospective, single-center design inherently limits causal inference and may reflect institutional practices and expertise that are not generalizable to other centers. In particular, this study was conducted in a high-volume center with extensive experience in complex aortic repair, and the results may therefore not be directly applicable to lower-volume centers or those with less specialized expertise. Second, anesthesia selection was not randomized and was based on clinical judgment, leading to substantial baseline differences between groups. In particular, patients treated under LA represented a markedly higher-risk cohort, with a greater proportion of emergency and ruptured presentations, advanced age, and higher comorbidity burden. Residual confounding due to unmeasured variables cannot therefore be excluded. Third, the relatively small number of patients treated under LA limits statistical power and results in a low event-per-variable ratio, precluding robust adjusted analyses or meaningful comparative inference. Furthermore, the marked imbalance in group sizes (25 vs. 334 patients) increases the risk of type II error and may result in unstable effect estimates, with potential overestimation or underestimation of observed associations, as reflected by wide confidence intervals. Accordingly, all outcome analyses should be considered exploratory and descriptive rather than confirmatory. Fourth, the study population was heterogeneous, including a mix of FEVAR, BEVAR, and combined procedures, which may influence both procedural complexity and outcomes. In addition, anesthesia and neuroprotective strategies were not standardized, with variability in the use of staged repair, MISACE, and cerebrospinal fluid drainage. Fifth, although conversion from LA to GA was recorded, the retrospective design limits detailed assessment of the timing and impact of conversion on outcomes. Sixth, outcomes were limited to the early postoperative period. As the primary aim of this study was to assess feasibility and early clinical outcomes, no conclusions can be drawn regarding long-term durability, late complications, or survival. Reinterventions were not further classified and likely represent a heterogeneous group of procedures. As such, interpretation of reintervention rates should be made with caution. Additionally, detailed causes of death were not consistently available due to the retrospective design. Finally, although SCI and neurological outcomes were recorded, standardized neuromonitoring protocols were not applied uniformly, and subtle or delayed neurological deficits may have been underreported.

## 6. Conclusions

LA appears technically feasible in carefully selected high-risk patients undergoing complex F/BEVAR procedures in experienced centers. However, given the substantial baseline and procedural differences between groups, this study does not allow conclusions regarding the comparative safety or efficacy of local versus general anesthesia. The observed differences in outcomes likely reflect variations in clinical presentation, procedural complexity, and use of adjunctive neuroprotective strategies. Careful patient selection, procedural planning, and the use of adjunctive strategies are essential for optimizing outcomes when LA is employed in complex aortic repair.

## Figures and Tables

**Table 1:** Baseline characteristics of a cohort of 359 patients treated with F/BEVAR.

Patient Demographics	LA *n* = 25 (%)	GA *n* = 334 (%)	*p* Value	SMD
Age	78.2 ± 9.6	72.1 ± 9.5	0.002	0.64
Age > 75 years	18 (72.0)	142 (42.5)	0.004	0.62
Male Sex	19 (76.0)	240 (71.9)	0.82	0.09
BMI	25.4 ± 3.6	25.7 ± 5.0	0.73	0.07
BMI ≥ 30	3 (12.0)	52 (15.6)	0.78	0.10
Hypertension	22 (88.0)	285 (85.3)	1.00	0.08
Dyslipidemia	14 (56.0)	211 (63.2)	0.47	0.15
Past Smoking	11 (52.4)	153 (46.8)	0.62	0.11
Current Smoking	5 (25.0)	106 (32.4)	0.49	0.16
Diabetes	3 (12.0)	50 (15.0)	1.00	0.09
ASA IV–V	14 (56.0)	69 (20.7)	<0.001	0.80
CKD	5 (21.7)	113 (35.3)	0.19	0.30
COPD	6 (25.0)	75 (23.4)	0.86	0.04
CHF	4 (16.7)	43 (13.4)	0.55	0.09
CAD	10 (41.7)	93 (29.0)	0.19	0.27
Arrhythmia	6 (25.0)	62 (19.4)	0.59	0.14
PAD	6 (25.0)	35 (10.8)	0.05	0.38
Prev. Stroke/TIA	3 (13.0)	26 (8.1)	0.43	0.16
History of Cancer	3 (15.0)	35 (10.9)	0.48	0.12
Connective Tissue Disorder	0 (0.0)	7 (2.2)	1.00	0.21
Previous Aortic Surgery	8 (33.3)	141 (44.1)	0.31	0.22
Previous Open AAA	1 (4.0)	10 (3.0)	0.78	0.06
Previous EVAR	7 (29.2)	35 (11.0)	0.02	0.47
Previous TEVAR	0 (0.0)	92 (28.9)	0.002	0.86

LA: Local Anesthesia; GA: General Anesthesia; SMD: Standardized Mean Differences; BMI: Body Mass Index; ASA: American Society of Anesthesiologists; CKD: Chronic Kidney Disease; COPD: Chronic Obstructive Pulmonary Disease; CHF: Chronic Heart Failure; CAD: Coronary Artery Disease; PAD: Peripheral Arterial Disease; TIA: Transient Ischemic Attack; AAA: Abdominal Aortic Aneurysm; EVAR: Endovascular Aortic Repair; TEVAR: Thoracic Endovascular Aortic Repair. Values are presented as mean ± SD or median (IQR) for continuous variables and *n* (%) for categorical variables. Percentages are calculated using available-case denominators; therefore, denominators may vary between variables due to missing data. SMD values > 0.2 indicate meaningful imbalance.

**Table 2:** Procedural characteristics of patients undergoing F/BEVAR according to anesthesia type.

Procedural Characteristics	LA *n* = 25 (%)	GA *n* = 334 (%)	*p* Value	SMD
Type of Aneurysm			0.14	
Complex AAA	15 (60.0)	150 (44.9)		0.31
TAAA	10 (40.0)	184 (55.1)		0.31
Emergency Repair	16 (64.0)	92 (27.7)	<0.001	0.77
Rupture	13 (52.0)	35 (10.5)	<0.001	1.01
Aneurysm Diameter (mm)	64.5 (59.0–88.5)	60.0 (54.0–70.0)	<0.001	
Type of Endovascular Repair			<0.001	
FEVAR	4 (16.0)	186 (55.7)		0.87
BEVAR	18 (72.0)	125 (37.4)		0.75
F/BEVAR	3 (12.0)	23 (6.9)		0.18
Number of Target Vessel	4.2 ± 0.8	3.9 ± 0.5	0.018	0.46
Number of Target Vessel > 4	4 (16.0)	15 (4.5)	0.035	0.37
Use of T-Branch	8 (34.8)	44 (13.5)	0.01	0.52
Use of CMD	16 (66.7)	242 (73.3)	0.48	0.14
Additional Procedures	15 (78.9)	114 (37.0)	<0.001	0.92
Percutaneous Lower Access	21 (95.5)	314 (95.7)	1.00	0.01
Upper Access	12 (66.7)	112 (35.6)	0.008	0.65
Staged Repair	0 (0.0)	129 (40.2)	<0.001	1.04
MISACE	0 (0.0)	77 (23.1)	0.008	0.73
CSF drainage	1 (4.2)	91 (28.0)	0.01	0.70

LA: Local Anesthesia; GA: General Anesthesia; SMD: Standardized Mean Differences; AAA: Abdominal Aortic Aneurysm; TAAA: Thoracoabdominal Aortic Aneurysm; FEVAR: Fenestrated Endovascular Aortic Repair; BEVAR: Branched Endovascular Aortic Repair; CMD: Custom-made Device; MISACE: Minimally Invasive Segmental Artery Coil Embolization; CSF: Cerebrospinal Fluid. Values are presented as mean ± SD or median (IQR) for continuous variables and *n* (%) for categorical variables. Percentages are calculated using available-case denominators; therefore, denominators may vary between variables due to missing data. SMD values > 0.2 indicate meaningful imbalance.

**Table 3:** Perioperative outcomes and complications of patients undergoing F/BEVAR according to anesthesia type.

Outcomes	LA *n* = 25 (%)	GA *n* = 334 (%)	*p* Value	OR (95% CI)
Technical Success	24 (96.0)	315 (94.3)	1.00	1.43 (0.17–11.9)
Endoleak at Final Angiography	5 (20.8)	82 (25.9)	0.58	0.75 (0.27–2.05)
Immediate Intervention	0 (0.0)	10 (3.2)	1.00	
ICU stay (days)	2.0 (1.0–4.5)	2.0 (1.0–4.0)	0.42	
Length of Stay (days)	11 (7.5–22.0)	8.0 (6.0–15.0)	0.08	
Blood Loss (mL)	650.0 (450.0–1300.0)	600.0 (400.0–1050.0)	0.55	
Blood Transfusion (mL)	316.0 (0.0–723.0)	0.0 (0.0–500.0)	0.004	
Operation Time (min)	229.5 (188.0–257.5)	241.0 (199.5–295.5)	0.88	
Fluoroscopy Time (min)	50.9 (44.0–83.0)	67.0 (50.2–88.0)	0.11	
Contrast Dose (mL)	232.0 (165.0–280.5)	228.0 (172.0–300.0)	0.99	
In-Hospital Complications	17 (68.0)	130 (41.3)	0.009	3.33 (1.35–8.19)
Pneumonia	3 (12.5)	22 (7.3)	0.41	1.81 (0.50–6.56)
UTI	0 (0.0)	10 (3.3)	1.00	
Arrhythmia	0 (0.0)	6 (2.0)	1.00	
MI	1 (4.0)	12 (4.0)	1.00	
Acute Heart Failure	1 (4.2)	15 (4.4)	1.00	
Renal Failure	6 (25.0)	38 (13.1)	0.12	2.23 (0.80–6.17)
Spinal Cord Ischemia	6 (24.0)	26 (8.5)	0.02	3.74 (1.37–10.19)
30 days stroke/TIA	0 (0.0)	4 (1.4)	1.00	
Ischemic Enterocolitis	1 (4.2)	2 (0.7)	0.21	
Post-Implantation Syndrome	1 (4.5)	31 (10.2)	0.71	
Access Related Complications	4 (16.7)	30 (9.9)	0.30	1.82 (0.58–5.68)
In-hospital death	3 (12.0)	9 (2.9)	0.05	4.92 (1.24–19.47)
30-day Reintervention	7 (31.8)	31 (10.4)	0.009	4.02 (1.57–10.30)
30-day Type I or III endoleak	5 (22.7)	57 (19.0)	0.59	1.25 (0.47–3.30)

LA: Local Anesthesia; GA: General Anesthesia; OR: odds ratio; CI: confidence interval; ICU: Intensive Care Unit; UTI: Urinary Tract Infection; MI: Myocardial Infarction; TIA: Transient Ischemic Attack. Values are presented as mean ± SD or median (IQR) for continuous variables and *n* (%) for categorical variables. Percentages are calculated using available-case denominators; therefore, denominators may vary between variables due to missing data. Odds ratios were calculated for binary outcomes using available-case data. Estimates are not reported for variables with zero events in one group.

## Data Availability

The data presented in this study are available on request from the corresponding author.
